# Extracellular signal regulated kinase 5 and inflammasome in progression of mesothelioma

**DOI:** 10.18632/oncotarget.22968

**Published:** 2017-12-06

**Authors:** Joyce K. Thompson, Anurag Shukla, Alan L. Leggett, Phillip B. Munson, Jill M. Miller, Maximilian B. MacPherson, Stacie L. Beuschel, Harvey I. Pass, Arti Shukla

**Affiliations:** ^1^ Department of Pathology and Laboratory Medicine, Larner College of Medicine, University of Vermont, Burlington, VT 05405, USA; ^2^ Department of Cardiothoracic Surgery, Langone Medical Center, New York University, New York, NY 10012, USA

**Keywords:** mesothelioma, extracellular signal regulated kinase 5, inflammasome, asbestos, XMD8-92

## Abstract

Malignant mesothelioma is an aggressive cancer in desperate need of treatment. We have previously shown that extracellular signaling regulated kinase 5 (ERK5) plays an important role in mesothelioma pathogenesis using ERK5 silenced human mesothelioma cells exhibiting significantly reduced tumor growth in immunocompromised mice. Here, we used a specific ERK 5 inhibitor, XMD8-92 in various *in vitro* and *in vivo* models to demonstrate that inhibition of ERK5 can slow down mesothelioma tumorigenesis. First, we show a dose dependent toxicity of XMD8-92 to 2 human mesothelioma cell lines growing as a monolayer. We also demonstrate the inhibition of ERK5 phosphorylation in various human mesothelioma cell lines by XMD8-92. We further confirmed the toxicity of XMD8-92 towards mesothelioma cell lines grown as spheroids in a 3-D model as well as in intraperitoneal (immune-competent) and intrapleural (immune-deficient) mouse models with and without chemotherapeutic drugs. To ascertain the mechanism, we explored the role of the nod-like receptor family member containing a pyrin domain 3 (NLRP3) inflammasome in the process. We found XMD8-92 attenuated naïve and chemotherapeutic-induced inflammasome priming and activation in mesothelioma cells. It can thus be concluded that ERK5 inhibition attenuates mesothelioma tumor growth and this phenomenon in part is regulated by the inflammasome.

## INTRODUCTION

Malignant mesothelioma is an aggressive cancer of mesothelial cell linings of pleural, peritoneal or pericardial cavities and rarely of tunica vaginalis [1]. It is a locally invasive cancer with limited treatment strategies and there is a dire need to identify new therapeutic options in order to improve patients’ prognosis. Previously, our group described the involvement of extracellular signal regulated kinases (ERKs) in mesothelioma tumorigenesis [2, 3] and we were first to propose ERK5 as a potential therapeutic target for mesothelioma [4, 5]. Here, we demonstrated the effect of an ERK5 specific inhibitor in various *in vitro* and *in vivo* models including immune-competent mice and showed the role of the inflammasome in the process.

ERK5, while belonging to the family of ERKs, has many differences in its structure and function from ERK1/2. ERK5 has an extended c-terminus containing a nuclear localization signal (NLS) and two regions rich in proline and a transcriptional activation domain (TAD). Compared to its ERK family counterparts, these additional features makes its molecular weight twice as much as other mitogen activated protein kinases (MAPK) and ERK5 is suitably termed big a MAP Kinase 1 (BMK1). The ERK5 pathway is not as well studied in tumorigenesis as other MAPKs are, but its significance is slowly emerging. A recent review summarizes the tumorigenic properties of ERK5 in both *in vitro* and *in vivo* models, which highlights various approaches used to demonstrate the role of ERK5 in different types of cancers [6]. Further, findings from our group have characterized ERK5 as an important player in mesothelioma tumorigenesis and drug resistance [4, 5, 7]. Our previous results were generated by genetic manipulations of ERK5 in immune-deficient mice and in the present study we moved our research to the next step by using a specific small molecule inhibitor of ERK5 on mesothelioma tumor growth in immune competent mice.

Recently, a number of small molecule inhibitors specific for ERK5 attenuation have been made available [6]. For our purposes we used XMD8-92, described in detail elsewhere [8]. Briefly, XMD8-92 is a selective inhibitor of ERK5 phosphorylation that does not exert any effects on close family member MEK5 or ERK1/2. Pharmacokinetics and tolerability assessment of XMD8-92 by various exposure routes, including oral, intravenous and intraperitoneal, demonstrated moderate tissue distribution and good bioavailability by all routes. In addition, this inhibitor was found to be well tolerated without mortality and morbidity in rodents [8].

Mesothelioma is an inflammation driven cancer and inflammasomes play important roles in its development [9–13]. Thus, the inflammasome should be considered when designing therapeutic strategies as we have recently reported [12, 13]. The nod-like receptor family member containing a pyrin domain 3 (NLRP3) inflammasome, a multiprotein complex that has been shown to be activated by asbestos, which results in caspase-1 activation and secretion of matured interleukin-1β (IL-1β) and many other pro-inflammatory factors [10, 11]. ERK5 is also known to promote inflammation [14] and is a critical mediator of inflammation driven cancers [15]. We hypothesized that there is a link between ERK5 and inflammasomes in promoting mesothelioma tumorigenesis. To demonstrate this, we first showed that the ERK5 specific inhibitor, XMD8-92, is effective at inhibiting mesothelioma tumorigenesis in various *in vitro* and *in vivo* models. In addition, we demonstrated that ERK5 inhibition by XMD8-92 diminishes endogenous as well as chemotherapeutic-induced inflammasome activation in mesothelioma cells. Our findings may lead the way to designing a more effective combination therapy for mesothelioma treatment.

## RESULTS

### Human mesothelioma tumors show enhanced staining for activated ERK5 (pERK5) as compared to normal lung tissue

**Figure 1:**
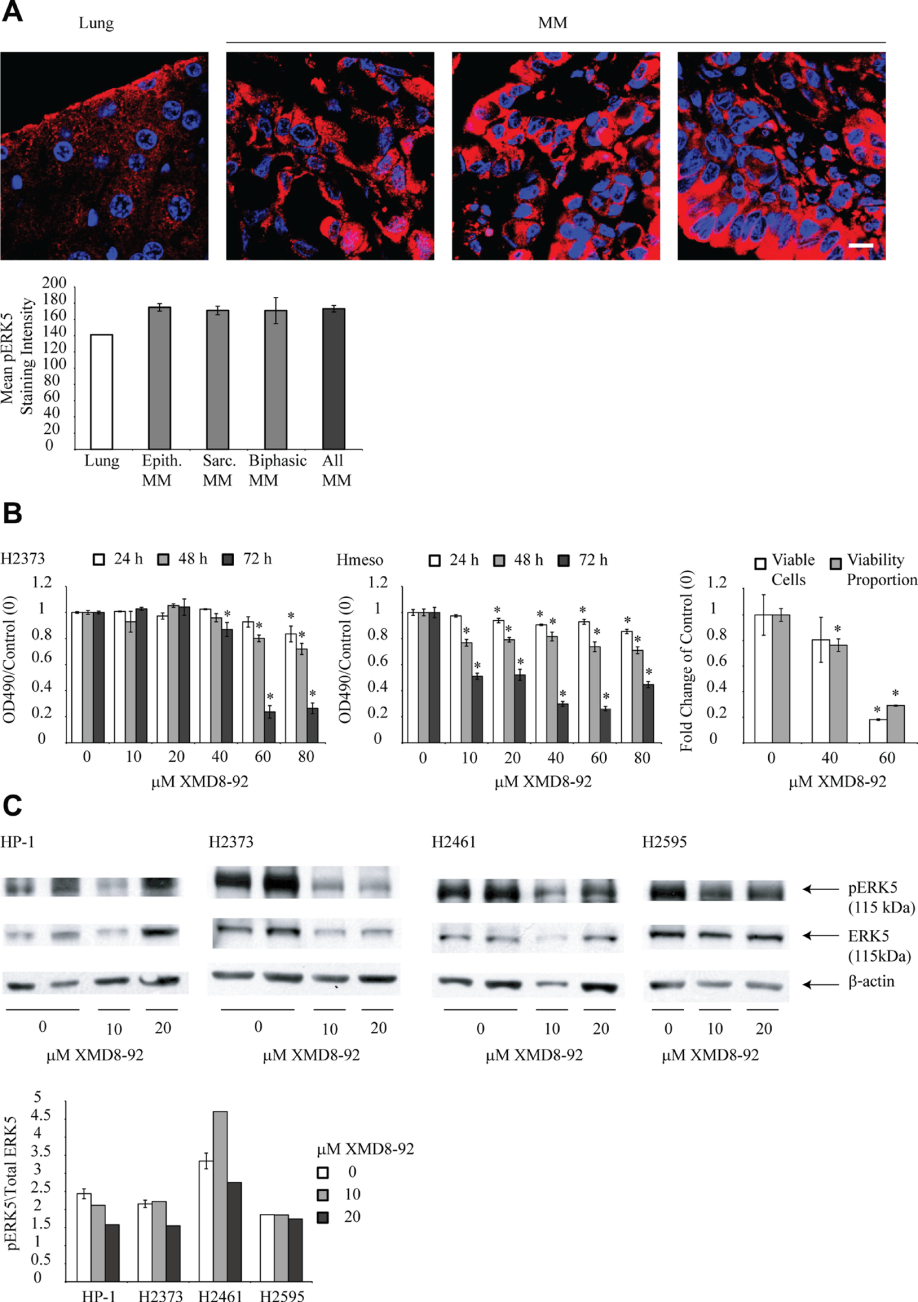
ERK5 inhibitor XMD8-92 is cytotoxic to human mesothelioma cells and inhibits constitutive ERK5 phosphorylation (**A**) Three representative human mesothelioma tumors, (out of 15 total) showing increased pERK5 (red) staining as compared to lung on the same slide, scale bar = 10 µm, nuclei stained blue. Below, quantitation of pERK5 intensity. (**B**) Human mesothelioma cell lines, H2373 and Hmeso were treated with various concentrations of XMD8-92 for different time points (*n* = 6). Controls (0) received vehicle DMSO. MTS assay was performed to assess cytotoxic effect of drug. ^*^p ≤ 0.05 (ANOVA) as compared to vehicle control at the same time point (0). Right, viability measured by Trypan Blue exclusion assay of XMD8-92 treated Hmeso cells at 72 h. ^*^p ≤ 0.05 (ANOVA) as compared to control (0). (**C**) Four human mesothelioma cell lines (HP-1, H2373, H2461 and H2595) expressing high constitutive levels of pERK5 were treated with 10 or 20 μM XMD8-92 for 24 h and cell lysates were subjected to immunoblotting using antibodies against pERK5, ERK5 or β-actin. Below, quantitation of blots showing reduced expression of pERK5 in response to XMD8-92. All histograms are presented as mean ± SEM.

Mesothelioma tumor tissue arrays including 15 sections from the tumors of individual pleural MM patient with different histology (9 epithelioid, 3 biphasic, 3 sarcomatoid) assessed by a pathologist as described before [16], were stained for pERK5 as described in the materials and methods section. Figure 1A shows extensive staining of pERK5 in mesothelioma tumor tissue (3 representative sections presented) as compared to normal lung tissue from the same array. Staining was predominantly cytoplasmic in all 3 sections. No correlation of pERK5 expression with histological type of tumor was observed (Figure 1A). This suggests enhanced constitutive phosphorylation of ERK5 in mesothelioma tumor tissues. We did not have benign pleural lesions available to compare, which is why we used normal lung as a comparing tissue.

### ERK5 inhibitor XMD8-92 is cytotoxic to mesothelioma cells

Two human mesothelioma cell lines, Hmeso (epithelioid) and H2373 (fibrosarcomatoid) were treated with various concentrations of XMD8-92 for different time periods and growth was assessed using the MTS assay. Hmeso cells demonstrated higher sensitivity to XMD8-92 (Figure 1B). For H2373 cells, there were significant cytotoxic effects from the inhibitor only at higher concentrations and later time points (Figure 1B). To confirm that XMD8-92 is cytotoxic to MM cells and not just arresting cell proliferation, Trypan Blue exclusion test was performed on Hmeso cells. As shown in Figure 1B, XMD8-92 treatment has significant toxicity to Hmeso MM cells. These two mesothelioma cell lines were selected for experiments as they form tumors in SCID mice and are used for *in vivo* experiments. Significantly effective doses from this experiment were selected for use in subsequent experiments.

### XMD8-92 inhibits ERK5 phosphorylation in human mesothelioma cells

Selected human mesothelioma cells lines showing constitutive phosphorylation of ERK5 (HP-1, H2373, H2461 and H2595) [4] were treated with 2 concentrations of XMD8-92 and pERK5 and total ERK5 levels were assessed by Western blot analysis. A trend of decrease in levels of pERK5 was observed in 3 cell lines after high doses of XMD8-92 treatment (Figure 1C).

### XMD8-92 inhibits mesothelioma spheroid growth in a 3-D model as well as on soft agar

**Figure 2:**
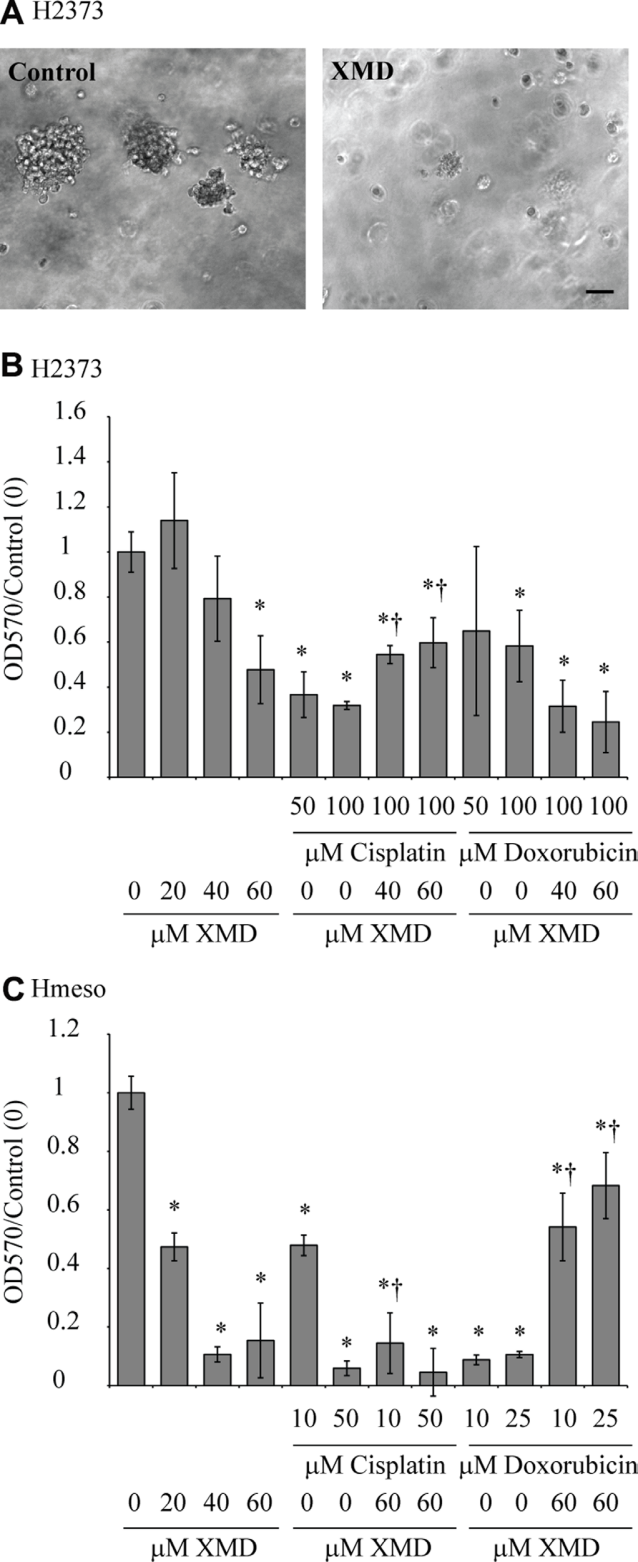
ERK5 inhibition attenuates mesothelioma colony formation as well as spheroid growth in a 3-D model (**A**) H2373 mesothelioma cells pretreated with XMD8-92 or vehicle were added to soft agar as described in the ‘materials and methods’ section (*n* = 6). A week later colonies were imaged, scale bar = 50 µm. (**B**) H2373 (*n* = 10) and (**C**) Hmeso cells (*n* = 6) were seeded in Cultrex 96-well plates from Trevigen as described in the ‘materials and methods’ section. Cells were grown with or without XMD8-92 in the presence and absence of chemotherapeutic drugs cisplatin or doxorubicin. MTS assay was performed on the 6th day. ^*^p ≤ 0.05 (*t*-test) as compared to untreated control; ^†^*p* ≤ 0.05 (*t*-test) as compared to drug alone. All histograms are presented as mean ± SEM.

To further test the effect of ERK5 inhibition on mesothelioma tumorigenesis, we grew H2373 cells pretreated with XMD8-92 on soft agar. As shown in Figure 2A, XMD8-92 pretreated cells formed very few and small colonies as compared to vehicle treated cells (control). Furthermore, XMD8-92 was also significantly effective in killing mesothelioma cells grown as a 3-D spheroid model (Figure 2B, 2C). In H2373 cells, combining doxorubicin and XMD8-92 lead to a greater decrease in cell growth (Figure 2B), however, this additive effect was not observed with the cisplatin/XMD8-92 combination. In Hmeso cells, the extent of cell killing was not increased by the addition of XMD8-92/cisplatin, maybe because the dose of XMD8-92 selected was very effective by itself. Surprisingly, the efficacy of doxorubicin was drastically reduced when combined with XMD8-92 in Hmeso cells which we were unable to explain (Figure 2C). Doses of doxorubicin or cisplatin used in this experiment were derived from a previously published report from our group [17]. Doxorubicin was used for combination studies here as this drug has been widely used as the most successful single agent for MM in past [18–21] and used currently in treatment of MMs.

### XMD8-92 inhibits mesothelioma tumor growth in mouse allograft (peritoneal) and xenograft (pleural) models

**Figure 3:**
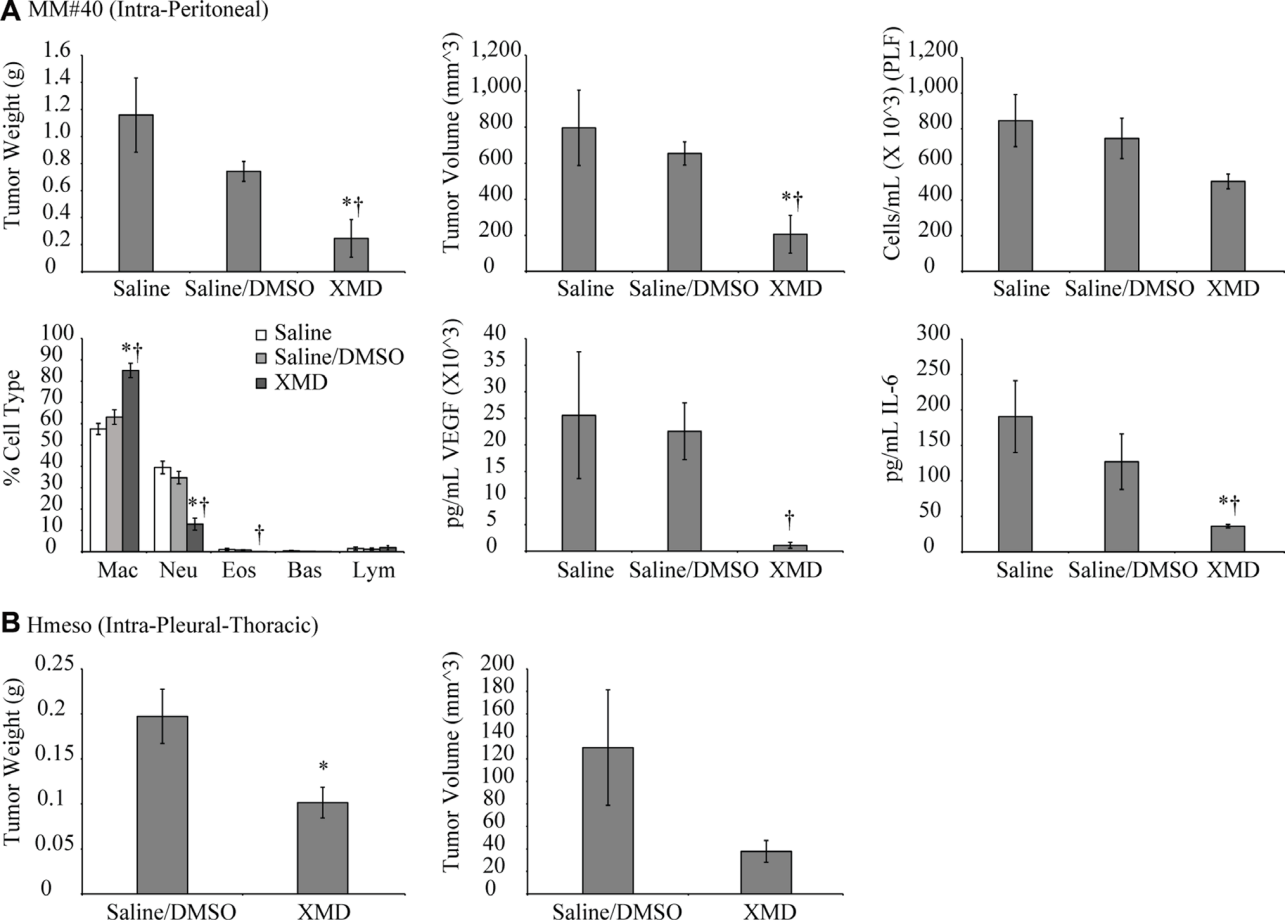
XMD8-92 attenuates mesothelioma tumor growth in peritoneal and pleural mouse models (**A**) Mouse mesothelioma cells #40 (2 × 10^6^) were injected intraperitoneally in C57/BL6 mice (*n* = 5/group). One week post cell injection XMD8-92 (50 mg/kg in saline:DMSO 50:50 solution) was injected intraperitoneally 1× daily for 3 weeks. Four weeks post cell injection tumors and peritoneal lavage fluid (PLF) were harvested and analyzed. ^*^p ≤ 0.05 (*t*-test) as compared to saline control; ^†^*p* ≤ 0.05 (*t*-test) as compared to saline:DMSO vehicle control. (**B**) Human mesothelioma cells Hmeso (2 × 10^6^) were injected into the pleural cavity of nude SCID mice (*n* = 8–15/group). One week later XMD8-92 (50 mg/kg) was injected intraperitoneally, 1× daily for 3 weeks. Four weeks post cell injection tumors were harvested from the pleural cavity and weight and volumes were assessed. ^*^p ≤ 0.05 (*t*-test) as compared to vehicle controls. All histograms are presented as mean ± SEM.

In the allograft model, mouse mesothelioma cells (MM#40) were injected IP, followed by XMD8-92 injection IP (50 mg/kg, 1× daily for 3 weeks). Four weeks post cell injection there was a significant reduction in tumor weights and volume as compared to vehicle control (saline: DMSO) (Figure 3A). While not significant, total cell numbers were reduced in PLF of the XMD8-92 group. Neutrophil numbers were significantly decreased in PLF of the XMD8-92 group as compared to controls (Figure 3A). Furthermore, levels of pro-inflammatory (IL-6) and angiogenic (VEGF) cytokine levels were also significantly reduced in PLF of XMD8-92 treated mice as compared to saline or vehicle treated mice (Figure 3A).

In the xenograft model, where human mesothelioma cells (Hmeso) were injected intrapleurally into nude-SCID mice and one week post cell injection XMD8-92 was administered IP daily, XMD8-92 showed effects on tumor reduction (Figure 3B) as compared to vehicle control. The effect of XMD8-92 on intrapleural tumors was less remarkable than in the intraperitoneal model (Figure 3A). This could possibly be due to local vs systemic effects of XMD8-92.

### ERK5 inhibition attenuates inflammasome priming and activation in mesothelioma cells

**Figure 4:**
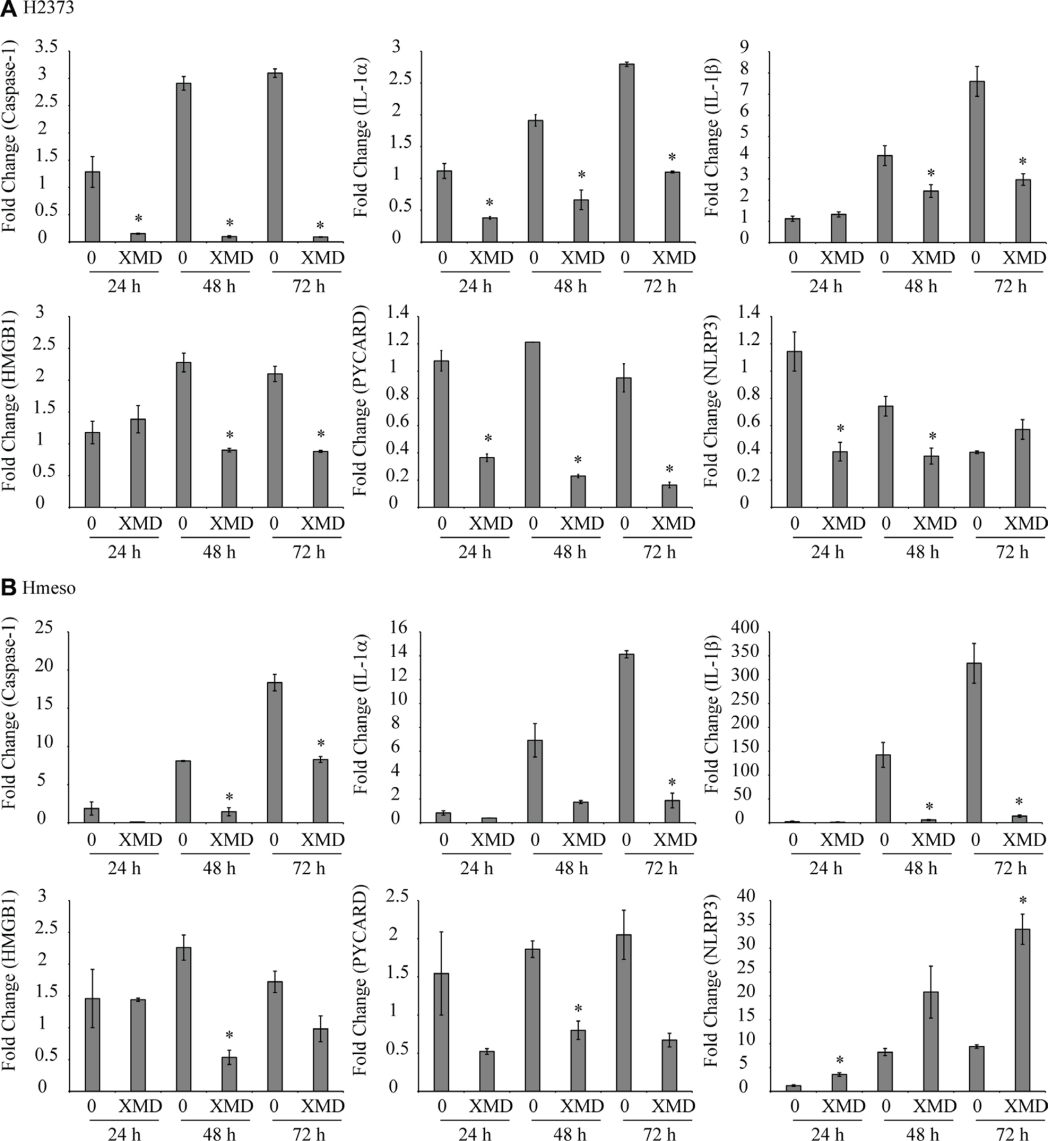
Endogenous expression of inflammasome-related genes were inhibited by XMD8-92 (**A**) H2373 and (**B**) Hmeso mesothelioma cells were treated with XMD8-92 (40 μM) for different periods of time (24, 48 or 72 h). Cells were harvested, RNA was extracted, cDNA synthesized and expression levels of various genes were assessed using qRT-PCR (*n* = 2). ^*^p ≤ 0.05 (*t*-test) as compared to vehicle control at the same time point (0). All histograms are presented as mean ± SEM.

**Figure 5:**
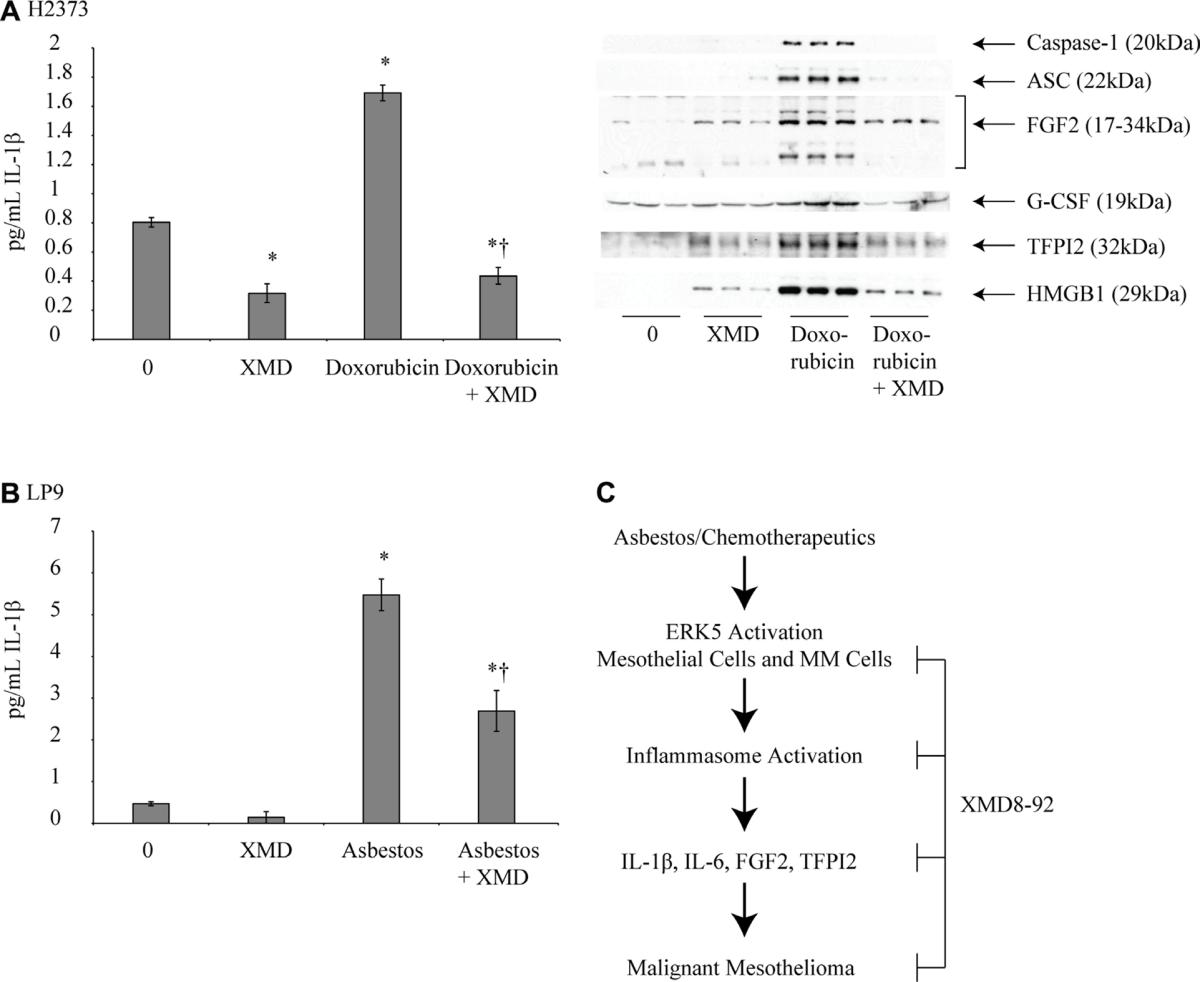
XMD8-92 inhibits doxorubicin-induced inflammasome activation in mesothelioma cells (**A**) Doxorubicin treatment (5 μM) in H2373 mesothelioma cells caused a significant increase in IL-1β secretion that was inhibited by pretreatment with XMD8-92 (*n* = 3). ^*=^ *p* ≤ 0.05 (ANOVA) as compared to vehicle treated control (0); ^†^= *p* ≤ 0.05 (ANOVA) as compared to doxorubicin alone group. Immunoblot panel showing various inflammasome related secretory proteins in conditioned medium after doxorubicin treatment with or without XMD8-92 pre-treatment. Due to secretory nature of proteins no normalization control could be included. (**B**) Human mesothelial cells (LP9) exposed to asbestos (5 μg/cm^2^) displayed increased IL-1β secretion that was inhibited by XMD8-92 pretreatment of cells (*n* = 2–4). ^*=^ *p* ≤ 0.05 (ANOVA) as compared to untreated control (0); ^†^= *p* ≤ 0.05 (ANOVA) as compared to asbestos alone group. All histograms are presented as mean ± SEM. (**C**) A schema showing that asbestos or chemotherapeutic drugs can cause ERK5 phosphorylation/activation in mesothelial or mesothelioma cells. ERK5 activation can result in inflammasome activation and cytokine and growth factor release which in turn can initiate mesothelioma development/progression/metastasis. ERK5 inhibitor XMD8-92 can block ERK5 phosphorylation and subsequent inflammasome activation and therefore mesothelioma progression.

Treatment of H2373 or Hmeso mesothelioma cells with XMD8-92 for varied time points (24–72 h) inhibited steady-state mRNA levels of constitutive inflammasome partners and related genes like *caspase-1, IL-1α*, *IL-1β*, *HMGB1* and *PYCARD* (encodes ASC protein) as compared to respective controls in both cell types (Figure 4A, 4B). *NLRP3* levels were decreased by XMD8-92 in H2373 at 24 and 48 h but increased in Hmeso cells at all time points, an interesting observation that needs detailed investigation (Figure 4A, 4B). In addition to endogenous levels of inflammasome related genes in mesothelioma cells, the doxorubicin-induced inflammasome activation (caspase-1 and ASC) and related cytokines and growth factors (IL-1β, FGF2, G-CSF, TFPI2 and HMGB1) secreted levels in medium were also blocked by XMD8-92 pre-treatment in H2373 cells (Figure 5A). No significant effect of XMD8-92 on doxorubicin-induced inflammasome activation in Hmeso cells was observed (data not shown). Furthermore, cisplatin-induced inflammasome activation was not attenuated by XMD8-92 (data not shown). Asbestos exposure is the cause of mesothelial to fibroblastic transition (MFT) and mesothelioma development and is known to activate ERK5 as well as the inflammasome in mesothelial cells [4, 11]. To understand if there is a connection between these two pathways, we tested the effect of XMD8-92 pretreatment on asbestos-induced IL-1β release in LP9 mesothelial cells. As shown in Figure 5B, XMD8-92 significantly inhibited asbestos-induced IL-1β release in mesothelial cells. ERK5 inhibitor XMD8-92 is also reported to block Tobacco smoke-induced urocystic EMT [22].

## DISCUSSION

ERK5 is an important signaling molecule known to play important roles in various cancers [6]. Based on *in vivo* and *in vitro* studies using genetically modified mesothelioma cell lines, our group was first to demonstrate that ERK5 plays a significant role in mesothelioma tumorigenesis and projected it as a potential therapeutic target [4]. With the development of a specific ERK5 inhibitor, XMD8-92 [8], it became possible to test this small molecule inhibitor in pre-clinical mesothelioma models, so that a potential therapy can be developed for this deadly cancer. Furthermore, we included an immune-competent mouse model in this study to ascertain the role of the immune system in the process of mesothelioma tumorigenesis and treatment. In addition, ERK5 is projected as a critical mediator of inflammation-driven cancer [15] and we have demonstrated a strong role of inflammasomes in mesothelioma tumor initiation [13] and therapy development [12]. As such, in the current project we also investigated a link between ERK5 and inflammasomes.

At first we studied the effect of XMD8-92 on mesothelioma cells and tumor toxicity. XMD8-92 showed cytotoxicity in mesothelioma cells using mono layers as well as in a 3-D spheroid model. Having significant cytotoxic effects in a 3-D spheroid model is encouraging, as this shows that the inhibitor is capable of penetrating the spheroids mimicking solid tumors. In the 3-D model of H2373 cells, a cumulative response was observed with doxorubicin. Although Hmeso mesothelioma cells showed increased toxicity with XMD8-92 as compared to H2373 cells in a mono layer and in a 3-D model, we did not see the similar combined response with doxorubicin and XMD8-92 in this cell line. In addition, cisplatin had no combined effect with XMD8-92 in any of the cell lines studied. As a part of assessing the effect of XMD8-92 on mesothelioma tumorigenesis, we also studied the colony forming activity of mesothelioma cells on soft agar. Inhibition of ERK5 resulted in significantly decreased colony formation, supporting an earlier observation we reported with mesothelioma cells where ERK5 was inhibited by shRNA [4]. Previously, XMD8-92 was reported to inhibit EGF-induced ERK5 phosphorylation in multiple tumor cell lines [8]. In support of this observation, various mesothelioma cell lines tested showed decreased ERK5 phosphorylation when treated with EGFR phosphorylation inhibitor (AG1478) [4]. Furthermore, XMD8-92 attenuated constitutive ERK5 phosphorylation in 3 out of 4 mesothelioma cell lines tested. Taken together these findings suggest that in mesothelioma cell lines, XMD8-92-induced ERK5 inhibition could in part be mediated by EGFR.

To further test the effects of ERK5 inhibition on mesothelioma tumor growth, we performed *in vivo* studies using orthotopic immune-deficient xenograft (pleural) and immune-competent allograft (peritoneal) models. Findings of these studies demonstrated that XMD8-92 could inhibit mesothelioma growth in both models, more so in the peritoneal model than in the pleural model. This difference in effect between these two models could be due to direct availability of drug in higher doses to peritoneal tumors than to pleural tumors, as the route of injection of drug in both models was IP. Previously, using ERK5 inhibited mesothelioma cell lines (shERK5), we have demonstrated that ERK5 inhibition in combination with chemotherapeutic drugs could have a cumulative effect on mesothelioma tumor growth [4]. However, in the present study no combined effect of cisplatin and XMD8-92 was observed on *in vivo* mesothelioma tumor growth (data not shown). This could be attributed to the sequence of administration of the two drugs or the location where the tumor is growing. For example, our previous *in vitro* experiments showed that ERK5 silenced mesothelioma cells retained more drug inside of the cells [4]. In contrast, if the drug is given before ERK5 inhibition (as in the present experiment), it can be cleared out faster without much effect on the tumors growth. Unlike our previously published model (peritoneal) [4], here we investigated combined drug treatment on pleural mesothelioma tumor growth. The drugs and tumors were in different compartments. Whatever may be the reason for not observing a combined effect of two drugs, it is clear from our data that the ERK5 inhibitor XMD8-92 has inhibitory effect on tumor growth in pleural as well as peritoneal models. Furthermore, another important finding from this experiment is that XMD8-92 is capable of inhibiting mesothelioma tumor growth in both immune-compromised as well as immune-competent mice. Extensive experiments need to be performed with more drugs like pemetrexed (a drug of choice for mesothelioma) to resolve the issue of combined drug treatment strategy for mesothelioma. In addition to tumor weight and volume, XMD8-92 also inhibited total cell numbers in PLF as well as neutrophil counts. In support, Finegan *et al.* [15] have also reported reduced neutrophil infiltration in response to tetradecanoyl phorbol acetate (TPA), at the epidermis of ERK5 deleted mice. Angiogenic (VEGF) and pro-inflammatory cytokine (IL-6) levels were also significantly reduced in the PLF of XMD8-92 treated mice as compared to controls. There is a possibility that these growth factors and cytokines are released from immune cells rather than tumor cells, however, these cells constitute the tumor microenvironment and affect tumor growth. Therefore, inhibition of these factors by XMD8-92 is a significant finding. These findings indicate that ERK5 may promote mesothelioma tumor growth by increasing inflammation/angiogenesis.

As reported by us [4] and other groups [6, 15], ERK5 appears to play a major role in controlling inflammation and angiogenesis. In this study, we focused our attention to understand the role of a special component of inflammation, the ‘inflammasome’ and its modulation by ERK5 in mesothelioma cells. Inflammation is a major contributor to mesothelioma tumorigenesis and we have shown that inflammasomes play an important role in initiation of asbestos-induced mesothelioma by causing MFT [13]. Furthermore, chemotherapeutics are shown to activate inflammasomes in mesothelioma cells and a combination of chemotherapeutics with IL-1 receptor antagonist has been demonstrated to be a better strategy for mesothelioma tumor reduction [12]. As chemotherapeutic drugs have also been shown to activate ERK5 in mesothelioma cells [4], it is possible that there is a link between ERK5 and inflammasomes. To demonstrate this, mesothelioma cells were treated with XMD8-92 [8], with or without chemotherapeutics and inflammasome priming and activation was assessed. XMD8-92 treatment attenuated various constitutive inflammasome related parameters in both mesothelioma cell lines. In Hmeso cells, NLRP3 transcript levels were increased with XMD8-92 treatment in a time-dependent manner, whereas, all other parameters were decreased by XMD8-92. This suggests that NLRP3 priming is differentially regulated by ERK5 in this mesothelioma cell line. In addition, doxorubicin-induced inflammasome activation in this cell line was not effectively or significantly attenuated by XMD8-92 (data not shown). This could probably be due to the fact that ERK5 is not constitutively activated in this cell line and only a high concentration (25 μM) of doxorubicin was able to activate ERK5, as reported previously [4]. XMD8-92 inhibited doxorubicin-induced activation of the NLRP3 inflammasome in H2373 mesothelioma cells as depicted by the absence of caspase-1 p20 and IL-1β in the medium after XMD8-92 treatment. Our previous work has also demonstrated the activation of caspase-1 by chemotherapeutic drugs in MM cells. Inhibition of caspase-1 by specific caspase-1 inhibitor had different effects on drug-induced caspase-1 activity and viability of Hmeso and H2373 cells [12]. H2373 cells showed a robust increase in caspase-1 activity with drugs, which is significantly inhibited by caspase-1 inhibitor. The role of caspase-1 is also reflected similarly on the viability of H2373 cells in response to drugs. Hmeso cells on the other hand showed only moderate increase in caspase-1 activity by chemotherapeutic drugs and no increase in cell viability in response to caspase-1 inhibitor. The observed effects of combination drug on 3D spheroid model of MM in the present study could partly be attributed to the inhibition of drug-induced caspase-1 activation by XMD8-92.

Regardless of these *in vitro* findings in Hmeso cells, XMD8-92 inhibited *in vivo* Hmeso mesothelioma tumor growth, suggesting involvement of other pathways and signaling molecules. The fact that neutrophil counts were lower in PLF after XMD8-92 treatment (Figure 3A) also suggests that the inflammasome may be inhibited in these mice, as inflammasome knockout mice have been shown to have a lower number of neutrophils in bronchoalveolar lavage fluid (BALF) after asbestos inhalation [10]. Furthermore, supporting findings indicate loss or inhibition of ERK5 by genetic manipulation or by XMD8-92 reduces the neutrophil infiltration into the epidermis of mice and therefore also tumor growth [15]. Authors of the study also demonstrated that ERK5 is responsible for caspase-1 activation and subsequent pro-IL-1β cleavage in both *in vitro* and *in vivo* systems of TPA-induced epidermis carcinogenesis [15]. As caspase-1 and IL-1β activation is an integral part of inflammasome functional machinery, Finegan’s [15] study clearly suggests a role of ERK5 in TPA-induced inflammasome regulation and supports our findings. We have previously shown that TPA can prime the NLRP3 inflammasome in human mesothelial cells [11].

In conclusion, our studies here show that the ERK5 inhibitor, XMD8-92 can play a significant role in reducing mesothelioma tumor growth in pleural as well as peritoneal models and the mechanism may involve inflammasome inactivation. Results were encouraging in both immune-compromised as well as immune-competent mouse models. ERK5 inhibition via small molecule inhibitors in combination with chemotherapeutic drugs may be the future strategy to target mesotheliomas. A very recent report published by Lin *et al.* [23] suggests that the effects observed by ERK5 inhibitors could in fact be off target effects. Keeping this in mind, new more specific inhibitors need to be tried as soon as they become commercially available. Further studies are also required to understand how ERK5 can regulate inflammasome transcription and/or activation.

## MATERIALS AND METHODS

### Cell culture and treatments

Human malignant mesothelioma cell lines HP-1, H2373, H2461 and H2595 were kind gifts from Dr. Harvey Pass (New York University, New York, NY) [24]. Hmeso cells were isolated by Reale and colleagues [25]. Human peritoneal mesothelial cells LP9/TERT-1 (LP9) cells were purchased from Brigham and Women’s Hospital, Harvard University, Boston, MA [26]. Cells were cultured as per previously reported procedures [4]. Mouse MM#40 cells were a kind gift from Dr. Agnes Kane (Brown University) and were cultured and used as previously described [27]. Cell lines were authenticated by short tandem repeat (STR) DNA fingerprinting using the Promega CELL ID System (Promega, Madison, WI). The STR profiles of human cells were found to be of human origin and did not match known DNA fingerprints in the Cell Line Integrated Molecular Authentication database (http://bioinformatics.istge.it/clima/) but will serve as a reference for future work. The first lot of ERK5 inhibitor, XMD8-92 was a generous gift from Dr. Nathanael Gray (Dana Farber Cancer Research Institute, Boston, MA) [8]. Subsequently it was purchased from TOCRIS Biosciences (Minneapolis, MN, 4132). Stock solutions were made in dimethyl sulfoxide (DMSO) and diluted as required. Doxorubicin was purchased from Sigma (St. Louis, MO, D1515) and cisplatin from Alfa Aesar (Ward Hill, MA, 10471). Doses of doxorubicin and cisplatin used here were calculated based on our previously published studies with reported mesothelioma cell lines (17). Controls received equal volumes of vehicle (≤0.1% DMSO) and were treated similarly. NIEHS reference sample of crocidolite asbestos was used here as previously reported [4].

### MTS assay to measure cytotoxicity

Cell viability of mesothelioma cells in response to XMD8-92 treatment was measured using the colorimetric MTS Assay, CellTiter 96 Aqueous One Solution Cell Proliferation Assay (Promega), as per the manufacturer’s instructions. Briefly, cells were seeded in 96 well plates and allowed to attach and grow for up to 72 h. Cells were then transferred into low serum containing medium for 24 h before XMD8-92 treatment. After defined time periods of treatments, MTS reagent was added and plates were incubated at 37°C for 3 h. MTS bioreduction by viable cells to a colored formazan product was measured at 490 nm by spectrophotometry.

### Trypan blue exclusion assay

To test cytotoxic vs proliferation arrest effect of XMD8-92, Hmeso MM cells were treated with two concentrations (40 µM and 60 µM) of XMD8-92 for 72 h. Adherent cells were trypsinized and diluted 50% in 0.4% Trypan Blue Solution in PBS (Sigma). Trypan Blue positive (non-viable) and negative (viable) cells were counted with a haemocytometer. Both the total number of viable cells as well as the viability proportion expressed as the fold change of control values was determined for each group.

### Immunohistochemistry

Human mesothelioma tissue arrays (obtained from Dr. Harvey Pass, NYU) were used for pERK5 staining. The array contained 15 mesothelioma sections from different patients with pleural mesothelioma. A section of normal lung was also part of the array stained for pERK5. Arrays were deparaffinized and after antigen retrieval, were blocked, washed and incubated overnight at 4°C with diluted pERK5 antibody (Cell Signaling Technology, Danvers, MA) as previously described [16]. For a negative control, one slide with all sections was stained as described, excluding primary antibody. After further washing, tissue specimens were incubated with a fluorescently conjugated secondary antibody, AlexaFluor^®^ 647 (Thermo Fisher, Grand Island, NY). Following nuclei staining with DAPI (Thermo Fisher), sections were mounted, coverslipped and imaged with a Zeiss 510 META laser scanning confocal microscope (Carl Zeiss, Thornwood, NY). For quantitation fluorescent images of pERK5 (red channel only) were analyzed in MetaMorph imaging software to determine the area of positive staining and average intensity in the field of view. From this data, the average intensity within the positively stained area of pERK5 was determined for each group.

### Western blot analysis

Mesothelioma cells were lysed in 4× sample buffer and boiled at 95°C for 15 minutes as previously described after XMD8-92 treatment for immunoblotting, [13]. For analysis of pERK5 and total ERK5 of cell lysates, 40 μg of protein was loaded on 10% SDS-PAGE gels to resolve proteins. Immunoblotting for pERK5/total ERK5 and β actin was performed on transferred proteins using the corresponding primary antibodies (Cell Signaling Technology). Densitometry analysis of blots was performed using Quantity One 1D Analysis Software (Bio-Rad, Hercules, California). The band intensity ratio of pERK5 relative to total ERK5 was measured. Western blot analysis of media supernatants was performed after concentration. Equal volumes of media supernatants were concentrated using StrataClean resin beads (Agilent Technologies, Santa Clara, CA) as previously reported [12]. An equal volume of 4× sample buffer was added to beads after aspirating the media and samples were boiled for 5 minutes at 95°C. Thereafter, 15 μL of each sample was resolved on a 15% SDS-PAGE gel and immunoblotted for inflammasome activation related proteins, caspase-1, apoptosis-associated speck-like protein containing a caspase-associated recruitment domain (ASC), fibroblast growth factor-basic (FGF2), granulocyte-colony stimulating factor (G-CSF), tissue factor pathway inhibitor 2 (TFPI2) and the danger-associated high mobility group box 1 protein (HMGB1) (Abcam, Cambridge, MA). Due to the secretory nature of proteins no normalization control could be included. Ponceau stained membrane showed equal loading of proteins.

### Quantitative real-time PCR

To determine the effect of XMD8-92 treatment on constitutive inflammasome related genes in mesothelioma cells, total RNA was extracted using the Qiagen RNeasy Plus Mini kit as per the manufacturer’s instructions (Qiagen, Germantown, MD). One μg of RNA from each sample was reversed transcribed using Promega AMV Reverse Transcriptase Kit (Promega) as previously reported [4]. Specific gene expression was quantified using a primer and probe mixture (Applied Biosystems) and 7700 Sequence Prism Detector (Perkin Elmer, Applied Biosystems) [4].

### ELISA

Media supernatants were concentrated in Amicon centrifugal filtration units with a molecular weight limit of 10 kDa (Millipore, Billerica MA) as described previously [12]. The levels of IL-1β secreted in response to doxorubicin or asbestos with and without XMD8-92 exposure were then measured using the Human Quantikine IL-1β/IL-1F2 Immunoassay (R&D Systems, Minneapolis, MN) ELISA kit, following the manufacturer’s directions. Values are expressed as picograms of IL-1β per milliliter of supernatant initially collected.

### Cytokine and growth factor analysis in peritoneal lavage fluid by Luminex^®^

A custom Magnetic Luminex^®^ Screening Assay (R&D Systems, Minneapolis, MN) including chemokine (C-X-C motif) ligand 1 (KC), vascular endothelial growth factor (VEGF), chemokine (C-C motif) ligand 5, regulated on activation, normal T cell expressed and secreted (RANTES), interleukin 33 (IL-33), FGF2 (basic), chemokine (C-C motif) ligand 2 (JE), interleukin 6 (IL-6), G-CSF, interleukin 1α (IL-1α) and receptor for advanced glycation end product (RAGE) was used to assay mouse peritoneal lavage fluid (PLF) supernatants according to the manufacturer’s protocol. A Bio-Rad Bioplex^®^ II automated magnetic wash station was used between steps to wash the 96 well plates. Samples were then read and analyzed using a Bio-Rad Bioplex^®^ System and Bioplex^®^ Manager 6.1 software (Bio-Rad, Hercules, California).

### 3-D model to grow mesothelioma spheroids

To determine the efficacy of XMD8-92 on mesothelioma cell spheroids grown in 3-D model, we used the Cultrex 3-D Spheroid Colorimetric Proliferation/Viability Assay from Trevigen, Inc. (Gaithersburg, MD). Mesothelioma cells were seeded at a density of 2,500/well following the manufacturer’s protocol and spheroids were allowed to grow for 72 h before commencement of treatments with XMD8-92 and/or doxorubicin or cisplatin. Six days later colorimetric analysis (MTT) was performed as stated in the manufacturer’s protocol.

### Colony formation assay

To assess the effect of XMD8-92 on mesothelioma cell colony formation capability, H2373 mesothelioma cells were grown as monolayer culture and exposed to XMD8-92 or vehicle for 24 h. Cells were later trypsinized and seeded onto CytoSelect 96-well cell transformation assay (Cell Biolabs Inc., San Diego, CA) following the manufacturer’s protocol. Wells were monitored for colony formation after 1 week in culture and imaged by phase contrast using an Olympus IX70 microscope (Olympus, Waltham, MA) [4].

### *In vivo* models for mesothelioma tumor growth

For the allograft (immune-competent) mouse model, mouse MM#40 cells (2 × 10^6^) [28] suspended in 50 μL saline were injected into the lower left quadrant of the peritoneal cavity of 7 week old, male C57/BL6 mice as previously described [27]. One week post cell injection, one group of mice (*n* = 5) started receiving XMD8-92 intraperitoneally (IP) (50 mg/kg in 1:1 mixture of saline and DMSO) once daily for 3 weeks. This dose of XMD8-92 was derived from a published report [8] and found to have no toxicity in mice. Two control groups of 5 mice each were also run simultaneously. One group received equal volumes of saline and other received a 1:1 mix of saline and DMSO via IP injections. Four weeks post cell injections, mice were euthanized as described previously [27] and PLF was collected from each animal as previously described [13] for the measurement of cytokines by Luminex^®^ and identification of inflammatory cells infiltrating the peritoneum with or without XMD8-92 treatment. Cells collected from PLF were used for cytospin preparation for differential cell counts after determination of total cell numbers and the supernatant was used for cytokine and growth factor assessment by Luminex^®^ [13].

In the xenograft (immune-deficient) model, human mesothelioma cells (Hmeso) (2 × 10^6^) were injected intrapleurally (into the pleural space of the thoracic cavity) into 7-week-old Fox Chase nude-SCID mice (Charles River Laboratories) (*n* = 8–15). One week post cell injection XMD8-92 (50 mg/kg, IP) was injected once daily for 3 weeks as described above. At four weeks post cell injection, tumors were harvested from the chest cavity, collected together, weighed and the volume was measured [4]. All experiments using mice were approved by the Institutional Animal Care and Use Committee (IACUC) at the University of Vermont, Larner College of Medicine (Burlington, VT).

### Statistical analysis

All experiments were performed in duplicate or triplicate and repeated at least twice. A one-way analysis of variance (ANOVA) followed by a Newman-Keuls procedure for adjustment of multiple pairwise comparisons or the student’s unpaired two-tailed *t*-test was applied to all data to establish the significance of observed differences between the various experimental groups. *p* ≤ 0.05 was considered significant. All statistical analyses were performed using the GraphPad Prism software program version 6.0 (GraphPad Software, La Jolla, CA).

## References

[1] Thompson JK, Westbom CM, Shukla A (2014). Malignant mesothelioma: development to therapy. J Cell Biochem.

[2] Shukla A, Hillegass JM, MacPherson MB, Beuschel SL, Vacek PM, Pass HI, Carbone M, Testa JR, Mossman BT (2010). Blocking of ERK1 and ERK2 sensitizes human mesothelioma cells to doxorubicin. Mol Cancer.

[3] Shukla A, Hillegass JM, MacPherson MB, Beuschel SL, Vacek PM, Butnor KJ, Pass HI, Carbone M, Testa JR, Heintz NH, Mossman BT (2011). ERK2 is essential for the growth of human epithelioid malignant mesotheliomas. Int J Cancer.

[4] Shukla A, Miller JM, Cason C, Sayan M, MacPherson MB, Beuschel SL, Hillegass J, Vacek PM, Pass HI, Mossman BT (2013). Extracellular signal-regulated kinase 5: a potential therapeutic target for malignant mesotheliomas. Clin Cancer Res.

[5] Sayan M, Shukla A, MacPherson MB, Macura SL, Hillegass JM, Perkins TN, Thompson JK, Beuschel SL, Miller JM, Mossman BT (2014). Extracellular signal-regulated kinase 5 and cyclic AMP response element binding protein are novel pathways inhibited by vandetanib (ZD6474) and doxorubicin in mesotheliomas. Am J Respir Cell Mol Biol.

[6] Hoang VT, Yan TJ, Cavanaugh JE, Flaherty PT, Beckman BS, Burow ME (2017). Oncogenic signaling of MEK5-ERK5. Cancer Lett.

[7] Ramos-Nino ME, Blumen SR, Sabo-Attwood T, Pass H, Carbone M, Testa JR, Altomare DA, Mossman BT (2008). HGF mediates cell proliferation of human mesothelioma cells through a PI3K/MEK5/Fra-1 pathway. Am J Respir Cell Mol Biol.

[8] Yang Q, Deng X, Lu B, Cameron M, Fearns C, Patricelli MP, Yates JR, Gray NS, Lee JD (2010). Pharmacological inhibition of BMK1 suppresses tumor growth through promyelocytic leukemia protein. Cancer Cell.

[9] Mossman BT, Shukla A, Heintz NH, Verschraegen CF, Thomas A, Hassan R (2013). New insights into understanding the mechanisms, pathogenesis, and management of malignant mesotheliomas. Am J Pathol.

[10] Dostert C, Petrilli V, Van Bruggen R, Steele C, Mossman BT, Tschopp J (2008). Innate immune activation through Nalp3 inflammasome sensing of asbestos and silica. Science.

[11] Hillegass JM, Miller JM, MacPherson MB, Westbom CM, Sayan M, Thompson JK, Macura SL, Perkins TN, Beuschel SL, Alexeeva V, Pass HI, Steele C, Mossman BT (2013). Asbestos and erionite prime and activate the NLRP3 inflammasome that stimulates autocrine cytokine release in human mesothelial cells. Part Fibre Toxicol.

[12] Westbom C, Thompson JK, Leggett A, MacPherson M, Beuschel S, Pass H, Vacek P, Shukla A (2015). Inflammasome Modulation by Chemotherapeutics in Malignant Mesothelioma. PLoS One.

[13] Thompson JK, MacPherson MB, Beuschel SL, Shukla A (2017). Asbestos-Induced Mesothelial to Fibroblastic Transition Is Modulated by the Inflammasome. Am J Pathol.

[14] Wilhelmsen K, Xu F, Farrar K, Tran A, Khakpour S, Sundar S, Prakash A, Wang J, Gray NS, Hellman J (2015). Extracellular signal-regulated kinase 5 promotes acute cellular and systemic inflammation. Sci Signal.

[15] Finegan KG, Perez-Madrigal D, Hitchin JR, Davies CC, Jordan AM, Tournier C (2015). ERK5 is a critical mediator of inflammation-driven cancer. Cancer Res.

[16] Shukla A, Bosenberg MW, MacPherson MB, Butnor KJ, Heintz NH, Pass HI, Carbone M, Testa JR, Mossman BT (2009). Activated cAMP response element binding protein is overexpressed in human mesotheliomas and inhibits apoptosis. Am J Pathol.

[17] Hillegass JM, Blumen SR, Cheng K, MacPherson MB, Alexeeva V, Lathrop SA, Beuschel SL, Steinbacher JL, Butnor KJ, Ramos-Nino ME, Shukla A, James TA, Weiss DJ (2011). Increased efficacy of doxorubicin delivered in multifunctional microparticles for mesothelioma therapy. Int J Cancer.

[18] Isobe H, Wellham L, Sauerteig A, Sridhar KS, Ramachandran C, Krishan A (1994). Doxorubicin retention and chemoresistance in human mesothelioma cell lines. Int J Cancer.

[19] Bowman RV, Manning LS, Davis MR, Robinson BW (1991). Chemosensitivity and cytokine sensitivity of malignant mesothelioma. Cancer Chemother Pharmacol.

[20] Scherpereel A, Berghmans T, Lafitte JJ, Colinet B, Richez M, Bonduelle Y, Meert AP, Dhalluin X, Leclercq N, Paesmans M, Willems L, Sculier JP (2011). Valproate-doxorubicin: promising therapy for progressing mesothelioma. A phase II study. Eur Respir J.

[21] Saxena A, Chua TC (2009). Results of systemic pemetrexed-based combination chemotherapy versus cytoreductive surgery and hyperthermic intraperitoneal cisplatin and doxorubicin on survival in malignant peritoneal mesothelioma. Lung Cancer.

[22] Min J, Geng H, Liu Z, Liang Z, Zhang Z, Xie D, Wang Y, Zhang T, Yu D, Zhong C (2017). ERK5 regulates tobacco smokeinduced urocystic epithelialmesenchymal transition in BALB/c mice. Mol Med Rep.

[23] Lin EC, Amantea CM, Nomanbhoy TK, Weissig H, Ishiyama J, Hu Y, Sidique S, Li B, Kozarich JW, Rosenblum JS (2016). ERK5 kinase activity is dispensable for cellular immune response and proliferation. Proc Natl Acad Sci U S A.

[24] Pass HI, Stevens EJ, Oie H, Tsokos MG, Abati AD, Fetsch PA, Mew DJ, Pogrebniak HW, Matthews WJ (1995). Characteristics of nine newly derived mesothelioma cell lines. Ann Thorac Surg.

[25] Reale FR, Griffin TW, Compton JM, Graham S, Townes PL, Bogden A (1987). Characterization of a human malignant mesothelioma cell line (H-MESO-1): a biphasic solid and ascitic tumor model. Cancer Res.

[26] Dickson MA, Hahn WC, Ino Y, Ronfard V, Wu JY, Weinberg RA, Louis DN, Li FP, Rheinwald JG (2000). Human keratinocytes that express hTERT and also bypass a p16(INK4a)-enforced mechanism that limits life span become immortal yet retain normal growth and differentiation characteristics. Mol Cell Biol.

[27] Miller JM, Thompson JK, MacPherson MB, Beuschel SL, Westbom CM, Sayan M, Shukla A (2014). Curcumin: a double hit on malignant mesothelioma. Cancer Prev Res (Phila).

[28] Goodglick LA, Vaslet CA, Messier NJ, Kane AB (1997). Growth factor responses and protooncogene expression of murine mesothelial cell lines derived from asbestos-induced mesotheliomas. Toxicol Pathol.

